# Evolution of *Bordetella pertussis* in the acellular vaccine era in Norway, 1996 to 2019

**DOI:** 10.1007/s10096-022-04453-0

**Published:** 2022-05-11

**Authors:** Lin T. Brandal, Didrik F. Vestrheim, Torbjørn Bruvik, Ragnhild B. Roness, Martha L. Bjørnstad, Margrethe Greve-Isdahl, Anneke Steens, Ola B. Brynildsrud

**Affiliations:** 1 Norwegian Institute of Public Health, Oslo, Norway; 2 European Program for Public Health Microbiology Training (EUPHEM), European Centre for Disease Prevention and Control (ECDC), Stockholm, Sweden; 3 Norwegian University of Life Sciences, Ås, Norway

**Keywords:** *Bordetella pertussis*, Whooping cough, Whole genome sequencing, Evolution, Genomic variation, Vaccine-related antigens

## Abstract

We described the population structure of *Bordetella pertussis* (*B. pertussis*) in Norway from 1996 to 2019 and determined if there were evolutionary shifts and whether these correlated with changes in the childhood immunization program. We selected 180 *B. pertussis* isolates, 22 from the whole cell vaccine (WCV) era (1996–1997) and 158 from the acellular vaccine (ACV) era (1998–2019). We conducted whole genome sequencing and determined the distribution and frequency of allelic variants and temporal changes of ACV genes. Norwegian *B. pertussis* isolates were evenly distributed across a phylogenetic tree that included global strains. We identified seven different allelic profiles of ACV genes (A–F), in which profiles A1, A2, and B dominated (89%), all having *pertussis toxin* (*ptxA*) allele 1, *pertussis toxin promoter* (*ptxP*) allele 3, and *pertactin* (*prn*) allele 2 present. Isolates with *ptxP1* and *prn1* were not detected after 2007, whereas the *prn2* allele likely emerged prior to 1972, and *ptxP3* before the early 1980s. Allele conversions of ACV genes all occurred prior to the introduction of ACV. Sixteen percent of our isolates showed mutations within the *prn* gene. ACV and its booster doses (implemented for children in 2007 and adolescents in 2013) might have contributed to evolvement of a more uniform *B. pertussis* population, with recent circulating strains having *ptxA1*, *ptxP3*, and *prn2* present, and an increasing number of *prn* mutations. These strains clearly deviate from ACV strains (*ptxA1*, *ptxP1*, *prn1*), and this could have implications for vaccine efficiency and, therefore, prevention and control of pertussis.

**Supplementary Information:**

The online version contains supplementary material available at 10.1007/s10096-022-04453-0.

## Background

Pertussis, caused by *Bordetella pertussis* (*B. pertussis*), is a highly contagious acute respiratory infection. The disease affects people of all ages, but is especially severe in infants. Although pertussis is a vaccine-preventable disease with high vaccine coverage worldwide, a global resurgence of this infection has been observed [1]. Pertussis is endemic in many countries, with epidemic cycles occurring every 2–5 years [2]. In 2014, 24.1 million pertussis cases were estimated globally, with 160,700 deaths from pertussis in children less than 5 years of age [3]. In Europe, a notification rate of 8.2 cases per 100,000 population was reported in 2018, with the highest notification rate in infants followed by 10–14-year olds [4]. In Norway, a notification rate of 47.4 cases per 100,000 population was reported in 2019 (www.msis.no). Norway has, since 2011, consistently reported the highest notification rate of pertussis in all of Europe, and in contrast to the majority of European countries, adults (≥ 18 years of age) accounted for the majority of cases (55%) [5].

During the mid-1990s and in the early twenty-first century, whole cell vaccines (WCVs) were replaced by acellular pertussis vaccines (ACVs) in high-income countries, including Norway, where ACVs were introduced from 1998 [2]. To combat the increasing incidence of pertussis, Norway and other European countries implemented childhood and adolescent ACV boosters [6, 7]. In Norway, ACV boosters were introduced for 7-year olds in 2006/2007 and for 15-year olds in 2013/2014. Recently, maternal immunization was introduced in some countries to prevent severe pertussis in infants, but has yet to be introduced in Norway [8].

Several factors are contributing to the re-emergence of pertussis, like improved diagnostics [9], increased awareness of the disease [10, 11], decreased vaccine efficacy [12], waning immunity [8, 13, 14], and pathogen adaption [2, 15–19]. Growing evidence suggests that genetic divergence of circulating *B. pertussis* population away from vaccine antigens and the emergence of strains with increased pertussis toxin production might be contributing factors [17, 19–21]. Deficiency in pertactin (Prn), one of the components in ACVs, is widely distributed globally, and the rapid spread of Prn deficiency is likely vaccine driven [22]. Mutations in other ACV antigens such as pertussis toxin (*ptxA*), its promoter (*ptxP*), and fimbria (*fim2* and *fim3*) are also reported and shown to have quickly spread throughout the *B. pertussis* population [17, 23]. Currently, more than 90% of *B. pertussis* circulating in Europe have *ptxA1*, *ptxP3*, and *prn2* genotypes and the recent replacement of *ptxP1* with *ptxP3* is associated with increased pertussis toxin production [17, 19, 24]. Comparative genomic analysis has demonstrated that worldwide transmission of new strains is rapid, and that the global population of *B. pertussis* is evolving in response to vaccine introduction, potentially enabling vaccine escape [17, 25]. All these changes raise concern, as ACV-induced selection pressure could potentially threaten vaccine efficacy [22, 26].

Pertussis has resurged in Norway with high notification rates since the late 1990s. Although lethal cases are very rare, pertussis is poorly controlled [7, 27]. No overview of the molecular epidemiological situation of *B. pertussis* in Norway exists. Only a few Norwegian isolates from the last decade have previously been included in European studies, showing that the *ptxA1*, *ptxP3*, and *prn2* genotypes are frequent and that Prn-deficient isolates are present among isolates examined [16, 18, 20, 28]. In Norway, the three-component ACVs used in the childhood immunization program and as booster vaccine for 15-year olds include vaccine antigens from Tohama I: *ptxA2*, *ptxP1*, *prn1*, and *fhaB1* (https://bigsdb.pasteur.fr/bordetella/), whereas the two-component vaccine used as a booster vaccine for 7-year olds harbors vaccine antigens from the “Pillemer (P134)” strain, with *ptxA2* present, but no information on the allelic variant of *fhaB* [29]. This suggests that the Norwegian *B. pertussis* population might have ACV antigen profiles evolving away from the antigens used in ACVs. The aim of this retrospective study was to characterize the population structure of *B. pertussis* in Norway over the period from 1996 to 2019 and determine whether there have been antigenic shifts in the wake of the introduction of ACVs. As such change may have implications for vaccine efficiency, the results can inform vaccine policy in Norway.

## Material and methods

### *B. pertussis *isolates

Clinical microbiological laboratories from each of five regions of Norway (Eastern Norway, Southern Norway, Western Norway, Northern Norway, and Trøndelag County) referred *B. pertussis* isolates or PCR-positive samples from clinical specimens to the National Reference Laboratory (NRL) for pertussis at the Norwegian Institute of Public Health (NIPH) on a voluntary basis. Before 2002, pertussis cases were diagnosed with culture and serological methods; however, from 2002, the use of PCR gradually increased, first among the youngest age groups, and from 2012, the majority of cases with pertussis in Norway were diagnosed by PCR. Stratified convenience sampling of 180 *B. pertussis* isolates from the microbiological biobank at the NRL was performed, taking into account the time of sampling (year), place of residence (five regions of Norway), and age of the case at the time of sampling (< 20 years or ≥ 20 years). We included eight and 14 isolates from 1996 and 1997, the WCV era, respectively, and a median of eight (range 5–10) isolates annually from 1998 to 2019, the ACV era (Table S1). No isolates or PCR-positive samples were received at the NRL for the years 2010 and 2011. Twenty-five of the isolates have previously been included in European Pertussis Surveillance studies [16, 18, 20, 28] (Table S1).

### Culture, DNA extraction, and whole genome sequencing

Approximately 60 PCR-positive clinical samples were cultured on charcoal agar with 40 µg/ml cephalexin and 250 µg/ml amphotericin B (made in-house at the NIPH) at 35℃ without CO_2_ under humid conditions for up to 10 days. Pure cultures were verified by matrix-assisted laser desorption ionization time-of-flight mass spectrometry (MALDI-TOF MS) (Daltonics) and stored in the microbiological biobank at − 70℃. Strains selected from the biobank were cultured on charcoal agar as described above for 24–72 h prior to genomic DNA extraction using QIAamp DNA Mini QIAcube kit (Qiagen) on QIAcube (Qiagen) following the manufacturer’s procedure. DNA concentration was measured using a Qubit fluorometer (Invitrogen, Thermo Fisher Scientific). DNA libraries were constructed with KAPA HyperPlus (Roche Life Science) with KAPA Unique Dual-Indexed Adapter kit (Roche Life Science), following the manufacturer’s instructions. Clean-up and size selection were performed using magnetic AMPure XP beads (Beckman Coulter). DNA was then quantified using a Qubit fluorometer, and the size of fragments was measured using an Agilent 4200 TapeStation system (Agilent Technologies). Paired-end reads (300 bp × 2) were produced on an Illumina MiSeq platform according to standard protocols using the MiSeq Reagent kits (v2 600 cycles; Illumina). Illumina data was deposited in the European Nucleotide Archive under the accession numbers ERS7736033 to ERS7736212. FastQC (Babraham Bioinformatics) was used for quality control of the raw reads. Sequence reads were trimmed and adapters removed using Trimmomatic v0.36. FASTA contigs were obtained from the trimmed reads using SPAdes v3.13.2. The final assembly files consisted of a median number of 277 contigs/sample (range: 261 to 1568 contigs) and had a median of 75.9 coverage/sample (range: 14.9 to 520.4 coverage). The mean length/sample was 3,885,772 bp. The *B. pertussis* genome is ~ 4.1 Mb, and the estimated mean coverage across all sequencing runs was 94.2%. Kraken (version 1.1.1) was used to assign taxonomic label species identification. All, except one sample, showed pure cultures of *B. pertussis*. The last sample was contaminated with *Streptococcus* (Table S1).

### Multilocus sequence typing

Multilocus sequence typing (MLST) according to the Institut Pasteur scheme (https://bigsdb.pasteur.fr/bordetella/) [30] was obtained using the program mlst v2.15 (https://github.com/tseemann/mlst) on the filtered FASTA contigs described above.

### Allelic variants of vaccine antigen genes and presence of molecular markers of erythromycin resistance

Sequences of allele variants of genes encoding vaccine antigens:* pertussis toxin* (*ptxA*, 39 alleles), *pertussis toxin promoter* (*ptxP*, 40 alleles), *pertactin* (*prn*, 140 alleles), *fimbriae 2* (*fim2*, 15 alleles), *fimbriae 3* (*fim3*, 38 alleles), and *filamentous hemagglutinin B* (*fhaB*, 100 alleles) were downloaded from the Institut Pasteur database at https://bigsdb.pasteur.fr/bordetella/. For assembled genomes, in silico typing of the vaccine antigen genes and screening for 23S ribosomal RNA (rRNA) A2037G (in Tohama I, NC_002929.2) mutation and the presence of erythromycin-resistant genes in the ResFinder database (2021–04-20) were performed using BLAST + tools (version 2.6.0) with strict criteria (100% match). Based on sequence similarities in Pasteur *prn* alleles 3 and 108, i.e., missing a 15-bp insertion (GGT CCC GGC GGC TTC at position 864) or having one single nucleotide polymorphism (SNP) (C > G at position 763) compared to *prn2*, respectively, these were interpreted as *prn2* to be comparable with previous publications (Table S1). Similarly, Pasteur *prn* allele 55, with one SNP (C > T) at position 2526 compared to *prn1*, was interpreted as *prn1*.

### Defining molecular mechanisms associated with mutations within the *prn* gene

A region including the *prn* gene was extracted from all 180 whole genome sequences (approximately 2748 bp). The sequences were imported and aligned in MEGA X [31] and manually examined for mutations using Tohama I (NC_002929.2), carrying *prn1*, as the reference. All sequences were screened for insertion sequence (IS) elements in *prn* using ISMapper (https://github.com/jhawkey/IS_mapper).

### Global *B. pertussis *sequence collection

Sequencing data of 371 publicly available *B. pertussis* strains isolated from cases identified in six different continents, Asia (*n* = 12), Africa (*n* = 2), North America (*n* = 300), South America (*n* = 8), Europe (*n* = 43), and Oceania (*n* = 5), were included in our study [32] (Table S2). The sequences were obtained both from long-sequence technology (PacBio) and short-sequence technology (Illumina). Complete genomes were available for 319 (86%) of the strains.

### SNP identification and phylogenetic analysis comparing the Norwegian *B. pertussis* population with the global population

After considering 319 publicly available closed *B. pertussis* genomes (Table S2), B1917 (accession: NZ_CP009751.1) was chosen as the most appropriate reference genome in the SNP analysis, having the highest degree of genomic homology to the Norwegian isolates included in this study, as assessed with HarvestTools v1.2 (http://dx.doi.org/10.1186/s13059-014-0524-x). Notably, Tohama I, the vaccine strain, was more distantly related to the Norwegian isolates than nearly every other publicly available *B. pertussis* genomes. In order to identify SNPs, reads were mapped to the B1917 genome using the Snippy pipeline v4.6.0 (https://github.com/tseemann/snippy). Maximum likelihood phylogenetic tree was created using iqtree v2.1.2 (https://doi.org/10.1093/molbev/msu300; https://doi.org/10.1038/nmeth.4285) with automatic model selection.

### Time-measured evolutionary analysis

Phylogenetic tree scaled to time was created using the program treetime v0.8.1 with the following non-default options: least-squares rerooting, accounting for covariation, and coalescent rate set to constant. The *R*^2^ value was 0.63. Figures were annotated using ITOL [33].

## Results

### Allelic profiles of ACV gene variants and the presence of molecular markers of erythromycin resistance

**Fig. 1:**
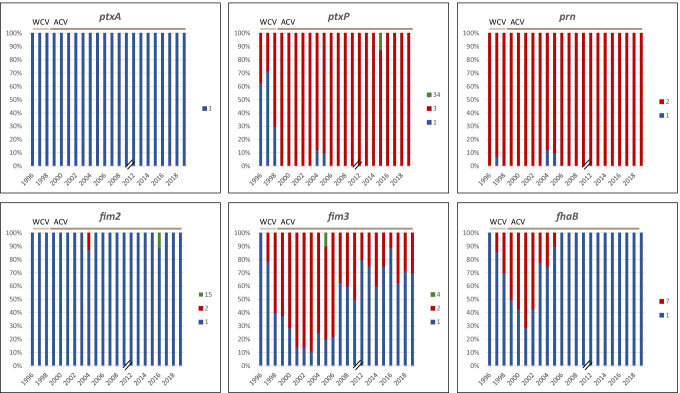
Distribution of allelic variants of the genes encoding acellular vaccine (ACV) antigens in the Norwegian *B. pertussis* population (*n* = 180), 1996–2019. The alleles *ptxA1*, *ptxP3*, *prn2*, *fim2-1*, and *fhaB1* were dominating in our isolates, whereas a more even distribution of the *fim3-1* and *fim3-2* alleles was seen. *ptxP1* was more common prior to introduction of ACV, whereas *ptxP3* dominated thereafter. Whole cell vaccine (WCV) period: 1996 and 1997; ACV period: 1998–2019. For the years 2010 and 2011, no *B. pertussis* isolates or PCR-positive samples were received at the National Reference Laboratory for pertussis at the Norwegian Institute of Public Health. Alleles were assigned using the following database: https://bigsdb.pasteur.fr/bordetella/ (see text for details)

The majority of the Norwegian *B. pertussis* isolates showed sequence type (ST) 2 (96%, 172/180), whereas the remaining had ST83. All our isolates carried *ptxA*1. The *ptxP*3 allele was dominating in our isolates (88%, 159/180), even though in 1996 and 1997, prior to introduction of ACV in Norway, *ptxP1* was present in 63% (5/8) and 71% (10/14) of the selected isolates, respectively (Fig. 1). Ninety-eight percent (177/180) of our isolates harbored *prn2*, and they showed Pasteur *prn* allele 2 (*prn*2, 159/177), allele 108 (16/177), and allele 3 (2/177) (Table 1 and Table S1). The three remaining isolates (2%, 3/180) carried *prn1* (one of the isolates showed Pasteur *prn* allele 55). The majority of our *B. pertussis* isolates had the *fim2-1* allele present (99%, 178/180). One isolate harbored the *fim2-2* allele, whereas the remaining isolate carried *fim2-15* (Table S1). The *fim3-1* allele was present in 52% (94/180) of our isolates, whereas 47% (85/180) had *fim*3-2. The remaining isolate carried *fim*3-4 (Table S1). The *fim3-1* allele was dominating prior to introduction of ACV in Norway in 1998 but was also common from 2007 and forward (Fig. 1). The majority of the Norwegian *B. pertussis* isolates carried *fhaB1* (85%, 153/180), whereas 15% (27/180) had the *fhaB7* allele (G > A at position 948 compared to *fhaB1*) (Table S1 and Fig. 1).

**Table 1:** Molecular mechanisms leading to mutations within the *prn* gene of *B. pertussis* in Norway, 1996–2019

Strain	Year of isolation	Allelic profile	*prn* allele Pasteur^a^	*prn* interpretation^b^	*prn* mechanism	Position in *prn* gene^c^	Accession number
BORP-NIPH-053	2001	A2	2	2	Deletion in the start of *prn*	1–300 bp	ERS7736085
BORP-NIPH-073	2004	F	55	1	*IS481* insertion	1613, 1614	ERS7736105
BORP-NIPH-099	2007	B	2	2	C > T STOP^d^	1258	ERS7736131
BORP-NIPH-105	2007	B	2	2	C > T STOP^d^	1258	ERS7736137
BORP-NIPH-107	2008	B	2	2	C > T STOP^d^	1258	ERS7736139
BORP-NIPH-109	2008	B	2	2	C > T STOP^d^	1258	ERS7736141
BORP-NIPH-111	2009	B	2	2	C > T STOP^d^	1258	ERS7736143
BORP-NIPH-114	2009	B	2	2	C > T STOP^d^	1258	ERS7736146
BORP-NIPH-128	2013	B	2	2	C > T STOP	223	ERS7736160
BORP-NIPH-129	2013	B	2	2	C > T STOP	223	ERS7736161
BORP-NIPH-132	2013	A1	108	2	*IS481* insertion	1613, 1614	ERS7736164
BORP-NIPH-138	2014	B	108	2	*IS481* insertion	1613, 1614	ERS7736170
BORP-NIPH-140	2015	B	108	2	IS481 insertion	1613, 1614	ERS7736172
BORP-NIPH-144	2015	B	108	2	Deletion of *IS1663* and proximal *prn*	1–1456	ERS7736176
BORP-NIPH-146	2015	B	2	2	Deletion in the start of *prn*	1–735	ERS7736178
BORP-NIPH-150	2016	B*^e^	108	2	*IS481* insertion	1613, 1614	ERS7736182
BORP-NIPH-151	2016	B	108	2	*IS481* insertion	1613, 1614	ERS7736183
BORP-NIPH-157	2017	B	108	2	*IS481* insertion	1613, 1614	ERS7736189
BORP-NIPH-161	2017	B	108	2	Deletion of *IS1663* and proximal *prn*	1–1233	ERS7736193
BORP-NIPH-163	2017	A1	108	2	IS481 insertion	1613, 1614	ERS7736195
BORP-NIPH-164	2018	B	2	2	*IS481* insertion	241, 242	ERS7736196
BORP-NIPH-165	2018	A1	108	2	*IS481* insertion	1613, 1614	ERS7736197
BORP-NIPH-167	2018	B	2	2	*IS481* insertion	241, 242	ERS7736199
BORP-NIPH-171	2019	A1	108	2	*IS481* insertion	1613, 1614	ERS7736203
BORP-NIPH-174	2019	B	108	2	Deletion of *IS1663* and proximal *prn*	1–1456	ERS7736206
BORP-NIPH-177	2019	B	108	2	Deletion of IS1663 and proximal *prn*	1–1456	ERS7736209
BORP-NIPH-178	2019	B	108	2	Deletion of *IS1663* and proximal *prn*	1–1456	ERS7736210
BORP-NIPH-179	2019	B	108	2	Deletion of *IS1663* and proximal *prn*	1–1233	ERS7736211
BORP-NIPH-180	2019	B	108	2	Deletion of *IS1663* and proximal *prn*	1–1456	ERS7736212

^a^None of the isolates showed complete match with the specified *prn* allele due to mutations; however, the Pasteur *prn* allele with the highest sequence homology was indicated (https://bigsdb.pasteur.fr/bordetella/)

^b^The *prn55* sequence has a SNP (C > T) at position 2526 compared to *prn1*, and *prn108* has a SNP (C > G) at position 763 compared to *prn2* (https://bigsdb.pasteur.fr/bordetella/), thus interpreted as *prn1* and *prn2*, respectively

^c^*prn* gene in Tohama I (NC_002929.2) used as a reference

^d^Confirmed the results of mutations in *prn* from a previous study[28]

^e^Allelic profile B*; BORP-NIPH-150 has *fim2-15* instead of *fim2-1*

Seven different allelic profiles were identified (profiles A1–F, Table 2). Profile A1 (*ptxA1*, *ptxP3*, *prn2*, *fim2-1*, *fim3-2*, *fhaB1*) and profile A2: identical to A1, except for replacement of *fhaB1* with *fhaB7*, occurred in 32% (58/180) and 15% (27/180) of the Norwegian *B. pertussis* isolates, respectively. Profile B (*ptxA1*, *ptxP3*, *prn2*, *fim2-1*, *fim3-1*, and *fhaB1*) was present in 42% (75/180), including one isolate with *ptxP34* instead of *ptxP3* and another isolate with *fim2-15* instead of *fim2-1* (both indicated as profile B* in Table S1). Profile C (*ptxA1*, *ptxP1*, *prn2*, *fim2-1*, *fim3-1*, and *fhaB1*) was seen in 9.4% (17/180) of the isolates. Two of the isolates had *prn3* instead of *prn2* (indicated as profile C* in Table S1). Profiles D–F were present in one isolate each, all carrying *ptxA1*, *ptxP1*, *prn1*, and *fhaB1*. These profiles had *fim2-1* or *fim2-2* and *fim3-1* or *fim3-4* (Table 2 and Table S1). Profile C was dominating prior to ACV implementation, whereas profile A2 was most common between 1998 and 2003 (Fig. S1). Profile A2 became rarer from around 2003 and was completely eradicated from the population by the time the ACV booster dose for children was introduced in 2006/2007. The period 2003–2007 also saw the relative growth of profile A1. From 2007, coinciding introduction of the second ACV booster dose for 15-year olds in 2013/2014, profile B was dominating in the Norwegian *B. pertussis* population (Fig. S1).

**Table 2:** Allelic profiles of acellular vaccine gene variants of *B. pertussis* in Norway, 1996–2019

Allelic profile	Acellular vaccine gene variants	No. of isolates
A1	*ptxA1*, *ptxP3*, *prn2*, *fim2-1*, *fim3-2*, *fhaB1*	58
A2	*ptxA1*, *ptxP3*, *prn2*, *fim2-1*, *fim3-2*, *fhaB7*	27
B^a^	*ptxA1*, *ptxP3*, *prn2*, *fim2-1*, *fim3-1*, *fhaB1*	75
C^b^	*ptxA1*, *ptxP1*, *prn2*, *fim2-1*, *fim3-1*, *fhaB1*	17
D	*ptxA1*, *ptxP1*, *prn1*, *fim2-1*, *fim3-4*, *fhaB1*	1
E	*ptxA1*, *ptxP1*, *prn1*, *fim2-1*, *fim3-1*, *fhaB1*	1
F	*ptxA1*, *ptxP1*, *prn1*, *fim2-2*, *fim3-1*, *fhaB1*	1

^a^Including one isolate with *ptxP34* instead of *ptxP3* and another isolate with *fim2-15* instead of *fim2-1* (both indicated as B* in Fig. 2 and Table S1)

^b^Including two isolates with *prn3* instead of *prn1* (both indicated as C* in Fig. 2 and Table S1)

None of the 180 *B. pertussis* isolates from Norway harbored mutations in the 23S rRNA gene but had wild type (A) at the SNP position A2037G (Tohama I, NC_002929.2). A single isolate from 2019 (allelic profile B) carried *msr(D)*, an ABC transporter involved in macrolide and streptogramin B resistance, and *mef(A)*, a marker for erythromycin and azithromycin resistance, respectively (Table S1).

### Molecular mechanisms involved in mutations within the *prn *gene

Sixteen percent (29/180) of the Norwegian *B. pertussis* isolates harbored mutations within the *prn* gene, including partial deletions and IS481 insertions (Table 1). Among these, 97% (28/29) carried *prn2* and one had *prn1*. Of the isolates with *prn* mutations, allelic profile B was dominating (79%, 23/29), whereas profile A1 occurred in four isolates (14%, 4/29) and profiles A2 and F in one isolate each, respectively. Twelve (41%) isolates had insertion of *IS481* in positions between 1613 and 1614 or between 241 and 242, including isolates with allelic profiles B (*n* = 7), A1 (*n* = 4), and F (*n* = 1). Eight (28%) isolates, all allelic profile B, had C > T mutation leading to stop codon at positions 1258 (*n* = 6) and 223 (*n* = 2), respectively. The last nine isolates (31%) had deletion in the start of *prn*, eight with allelic profile B and one with profile A2. Seven of the nine isolates had deletion of *IS1663* downstream of the *prn* gene including deletion of the proximal part of *prn* (Table 1). Mutations within the *prn* gene were more common from 2007, and in 2019, 60% (6/10) of the selected Norwegian *B. pertussis* isolates had *prn* mutations (Fig. S2).

### Evolution of *B. pertussis* in Norway from 1996 to 2019 and its correlation to alterations in pertussis vaccination

**Fig. 2:**
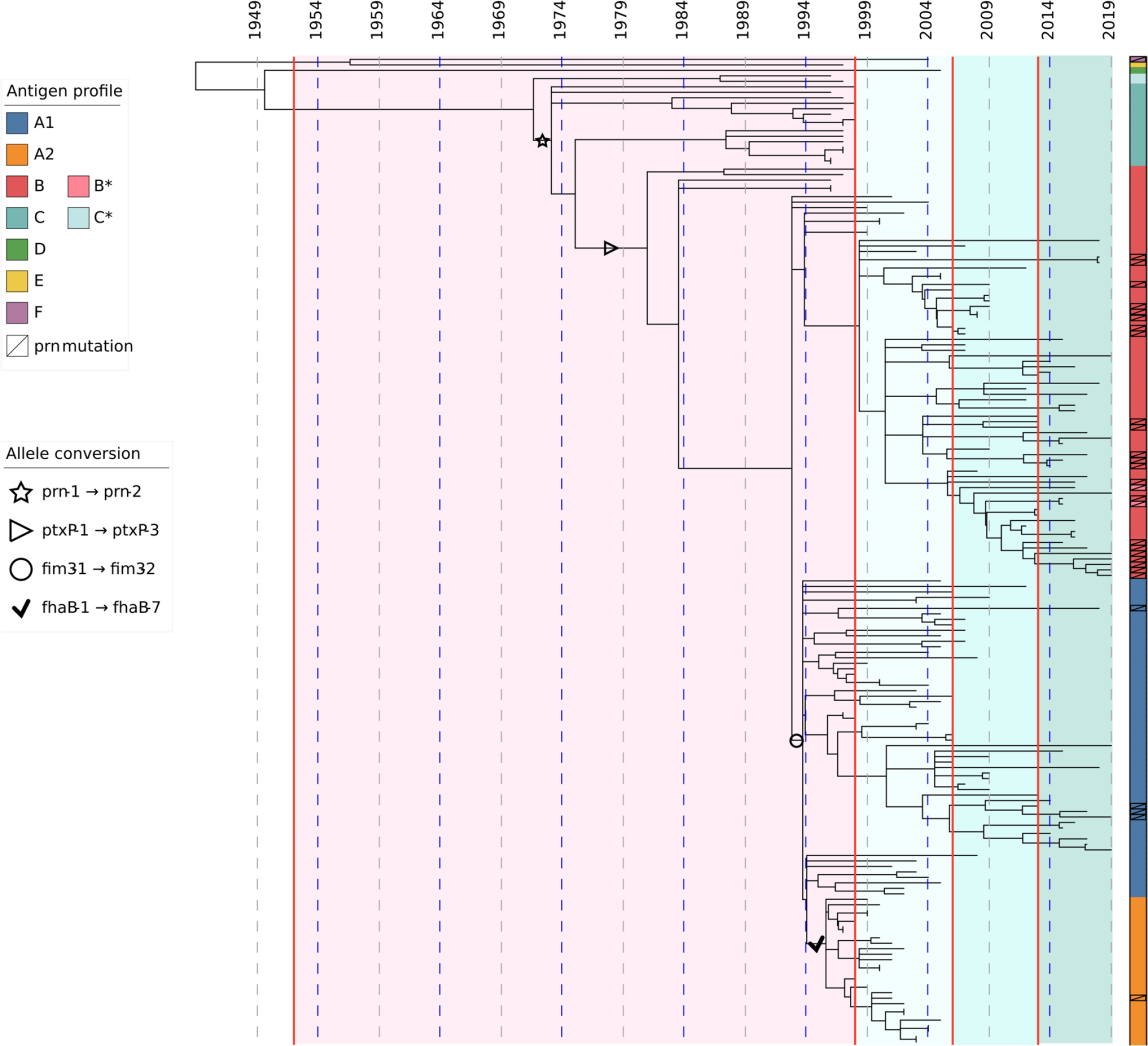
Molecular clock phylogeny of Norwegian *B. pertussis* isolates (*n* = 180), 1996–2019. Seven different allelic profiles of genes encoding acellular vaccine (ACV) antigens are indicated with different colors (A1, blue; A2, orange; B, red; C, turquoise; D, green; E, yellow; F, purple). Alterations of allele variants of the ACV antigens are shown as symbols (star, *prn1* → *prn2*; right-pointing triangle, *ptxP1* → *ptxP3*; circle, *fim3-1* → *fim3-2*; and black check mark, *fhaB1* → *fhaB7*). *B. pertussis* isolates with mutations within the *prn* gene compared to the *prn1* allele of Tohama I (NZ_002929.2) are indicated on the figure. The red vertical lines indicate implementation of the whole cell vaccine in Norway in 1952, the ACV in 1998, the first booster dose for 7-year olds in 2006, and the second booster dose for 15-year olds in 2013. The WCV era is indicated in pink color, whereas the ACV era is indicated in green. Antigen profile B* includes one isolate with *ptxP34* instead of *ptxP3* and another isolate with *fim2-15* instead of *fim2-1*. Antigen profile C* includes two isolates with *prn3* instead of *prn1*

Temporal changes of genes encoding ACV-related antigens showed that alterations of these genes occurred prior to introduction of ACV in 1998, but mainly after implementation of WCV in 1952 (Fig. 2). The *prn2* allele likely emerged prior to 1972 and the *ptxP3* allele before the early 1980s. *B. pertussis* isolates with allelic profile B (*ptxA1*, *ptxP3*, *prn2*, *fim2-1*, *fim3-1*, and *fhaB1*) were introduced around 1980, and allelic profile B is still the dominating profile of Norwegian *B. pertussis* isolates. *B. pertussis* isolates with allelic profiles C–F, all carrying *ptxP1* and *prn1*/*prn2*, were present before and during the WCV era, but disappeared around 2007. In the mid-1990s, the *fim3-2* allele appeared and allelic profile A1 (*ptxA1*, *ptxP3*, *prn2*, *fim2-1*, *fim3-2*, *fhaB1*) was introduced. After the mid-1990s, but before the introduction of ACV, allelic profile A2 occurred, carrying the *fhaB7* allele. However, profile A2 disappeared around 2005 prior to introduction of the first ACV booster dose. After 2005, only two allelic profiles (A1 and B) were present in the Norwegian *B. pertussis* population, both carrying *ptxA1*, *ptxP3*, *prn2*, *fim2-1*, and *fhaB1* and either *fim3-2* (A1) or *fim3-1* (B) (Fig. 2). An increase in the number of mutations within the *prn* gene was seen after the introduction of ACV and its booster doses, and these mutations were mainly seen in *B. pertussis* isolates with allelic profiles A1 and B (Table 1 and Fig. 2).

### Comparison of the Norwegian *B. pertussis* population with the global population

The available global *B. pertussis* isolates covered the time period 1936 to 2016 and 18 countries in six different continents, although 81% (300/370) of the isolates were from North America (Table S2). The majority of the isolates showed ST2.

**Fig. 3:**
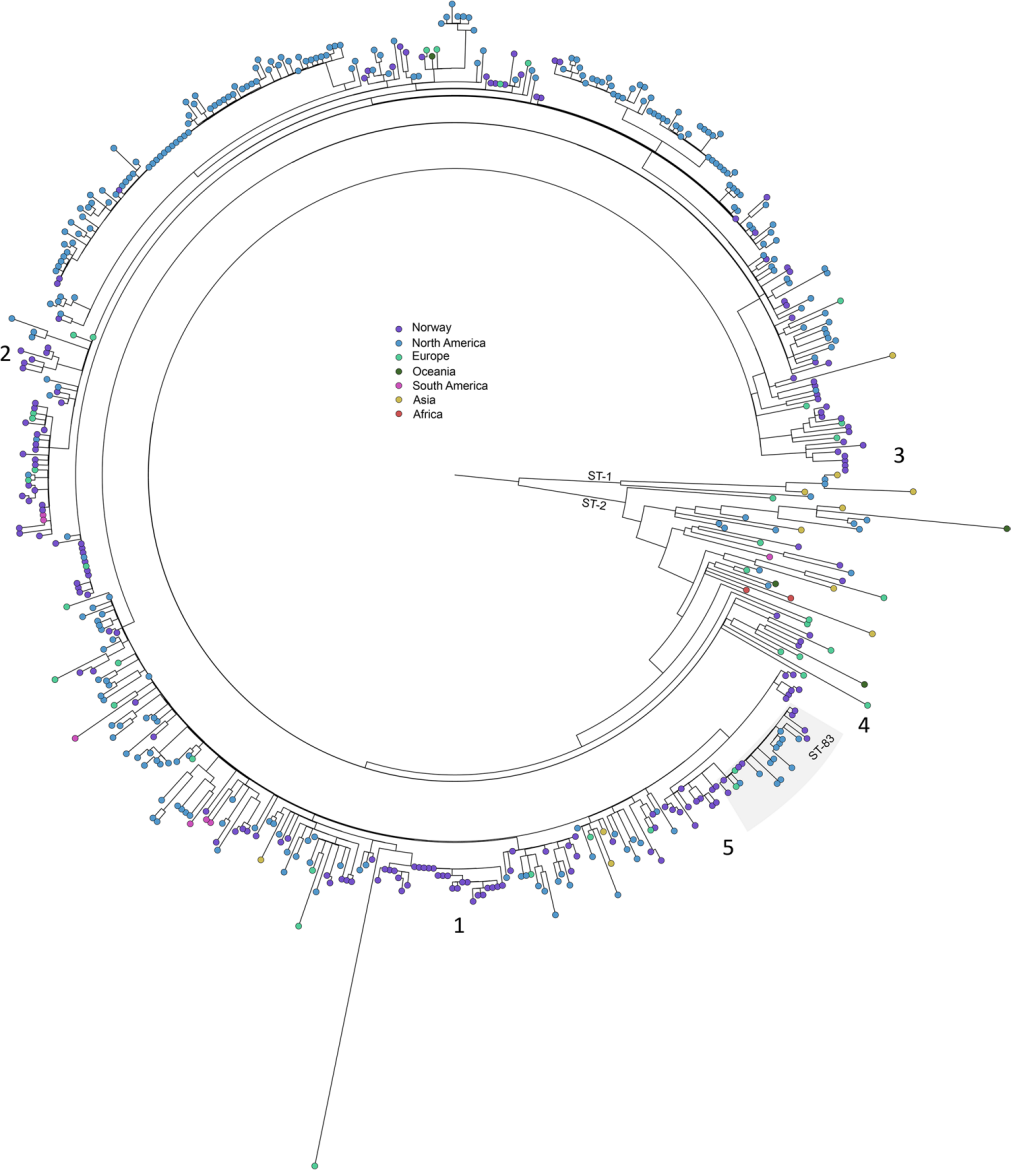
SNP-based phylogenetic tree of Norwegian *B. pertussis* isolates, 1996–2019, put in a global context. Norwegian strains (*n* = 180, purple circles) were distributed across the global phylogenetic tree; however, a few country-specific branches were detected (1–5). Worldwide strains (*n* = 370) were isolated from 1939 to 2016 and covered six different continents (Table S2)

The maximum likelihood tree showed that the Norwegian *B. pertussis* population was distributed across the global population of ST2 and ST83 isolates (Fig. 3). However, a few branches consisted entirely of Norwegian *B. pertussis* isolates. Branch 1 included isolates from 1997 to 2005, and they showed allelic profile A2, with the *fhaB7* allele present. Branch 2 consisted of a subgroup of Norwegian *B. pertussis* isolates with allelic profile B. These isolates were from 2012 to 2018, and the majority was from cases living in Eastern Norway. Branch 3 included *B. pertussis* from 1996 to 1998 carrying profile C, whereas branches 4 (1997–2006) and 5 (2009–2019) both consisted of isolates with allelic profile A1 (Fig. 3).

## Discussion

A global resurgence of pertussis has been seen after the introduction of ACVs in the 1990s [34, 35]. Changes in the *B. pertussis* population and pathogen adaptation have been suggested as one of the causes of this increase [2, 15, 17–19]. For Norway, little data was available on the *B. pertussis* population before this study and none of the isolates had previously been whole genome sequenced. The 180 whole genome–sequenced *B. pertussis* strains, isolated from 1996 to 2019, indicated clear molecular epidemiological shifts in the Norwegian population and that mutations in ACV antigens have occurred prior to and during the WCV (1952–1998) era. Our results further showed that ACV, introduced in 1998, and its two booster doses, implemented in 2006/2007 (7-year olds) and 2013/2014 (15-year olds), respectively, have led to a more uniform *B. pertussis* population, with two dominating allelic profiles (A1 and B) in recent years. Both profiles have the non-ACV alleles *ptxA1*, *ptxP3*, and *prn2* fixed and an increasing number of mutations within the *prn* gene.

In the Norwegian *B. pertussis* population, a heterogeneous mix of strains with a distinct time-dependent shift in allelic profiles was observed. The Norwegian strains were distributed across the global phylogenetic tree, supporting previous observations of rapid strain flow of *B. pertussis* between countries, even though a few country-specific branches were observed [17, 21, 25, 36, 37].

Temporal changes of genes encoding the ACV antigens in the Norwegian *B. pertussis* population were comparable with findings from other countries. The changes supported the evidence that genetic alterations are driven by the selective pressure imposed by vaccine-induced immunity, mainly from the WCV era, and accentuated in the ACV era [15, 17, 23, 38]. In concordance with the global population, *B. pertussis* with *ptxP3*, a gene variant leading to increased pertussis toxin production and associated with more severe disease in young infants, emerged prior to the early 1980s and dominated by the end of the 1990s [15, 17, 24, 36, 39]. The *prn2* allele emerged from the 1970s, as indicated by others, and is now the dominating *prn* allele in both WCVs and ACVs using countries, except for China and India [17, 19, 40–42]. Furthermore, the *fim3-2* allele appeared in the start of the 1980s and has since been present alongside *fim3-1* in the *B. pertussis* population worldwide [15, 18, 43].

Like other countries, we observed an increasing trend of mutations within the *prn* gene, including partial deletions and IS481 insertions, associated with time since implementing ACVs [16, 44, 45]. Even though Prn expression was not examined in our study, five of our isolates with *prn* mutations have previously been shown to be Prn-deficient [28]. Interestingly, few *prn*-negative isolates have been reported from countries still using WCVs as well as in countries without a Prn component included in their ACVs, indicating that this alteration is driven by ACVs [16, 19, 41, 42, 46–48]. This has clearly been demonstrated by Japan who implemented ACVs as early as 1981 followed by increased proportion of Prn-deficient strains from the mid-1990s. However, in 2012, Japan introduced ACVs without Prn and a decline in Prn-deficient strains was observed [19, 37, 47, 48].

During the WCV era, a number of antigenic allele conversions emerged, and the introduction of ACVs led to clonal expansions of strains with mutations that did not match the ACV profile. It has been shown that *B. pertussis* isolates with *ptxP3* and Prn expressed had increased fitness during the WCV era, whereas after implementing ACV, enhanced fitness was associated with Prn-deficiency, *ptxP3* and *fim3-1* [37]. Consequently, the diversity in allelic profiles decreased during the ACV era and approximately 90% of recent circulating *B. pertussis* isolates harbor *ptxA1*, *ptxP3*, and *prn2* with an increasing number of mutations within the *prn* gene [2, 21]. This demonstrates that the current *B. pertussis* population clearly deviates from the vaccine strains (*ptxA2*, *ptxP1*, *prn1*). This might affect the preventive potential of the ACVs and thus contribute to resurgence of pertussis [12, 38]. However, resurgence of pertussis is a complex phenomenon, and several other factors might favor the rise in pertussis disease. For instance, ACVs have been shown to be inferior to WCVs by not inducing mucosal immune response leading to increased colonization and transmission of *B. pertussis* and by inducing earlier waning immunity [12, 49]. Thus, novel ACVs with antigen targets matching the current circulating *B. pertussis* population or new targets, as well as appropriate adjuvants to stimulate both antibody and cell-mediated immune response, are highly warranted [49, 50].

Macrolide-resistant isolates are only sporadically seen in Europe, the Middle East, and North and South America [2]. This is in concordance with our findings, showing molecular determinants for macrolide resistance in only one *B. pertussis* isolate. This isolate carried the *msrD_2* and *mefA_10* genes, to our knowledge, never detected in *B. pertussis* previously; however, this sequence was contaminated with *Streptococcus*. Interestingly, in China, antibiotic abuse has led to a high level of erythromycin resistance in the *B. pertussis* population and contributed to the spread of *B. pertussis* lineages harboring *ptxA1*, *ptxP1*, and *prn1*, clearly deviating from the dominating clone seen in Europe, even though similar vaccine regimes were implemented both places years ago [32].

Limitations of the study include that the representativeness of our material can be questioned, because the primary laboratories in Norway submit *B. pertussis* samples to the NRL at the NIPH only on a voluntary basis and few isolates were included annually. Nonetheless, the similarities with previous studies indicate that our selection of isolates probably is representative for the Norwegian population. Second, we used short-sequence technology which is incapable of detecting rearrangements and large duplications responsible for the genome diversity seen in *B. pertussis* [51–53]. However, studies using long-sequence technology in combination with short sequencing technology show similar results to ours when it comes to temporal changes of ACV antigens [17, 21]. Finally, we have examined alterations at the genomic level and not at the transcriptomic or proteomic levels. The two latter might also be important for vaccine efficacy. Several countries have reported *B. pertussis* strains that do not express ACV antigens such as Prn, FhaB, and Ptx, although Prn deficiency is the only change frequently reported in the *B. pertussis* population [15, 19, 38, 54, 55].

## Conclusion

We observed molecular epidemiological shifts in the Norwegian *B. pertussis* population during the WCV and ACV eras. ACV use and its booster doses have led to lineage-specific proliferation of more successful, ACV-deviating strains, leading to a more uniform *B. pertussis* population. These clones have the *ptxP3* and *prn2* alleles fixed and are characterized by increasing incidence of mutations within the *prn* gene. The emergence of *B. pertussis* isolates with ACV antigens deviating from the vaccine antigens might have implications for vaccine efficiency and therefore prevention and control of pertussis. We recommend continued collection of *B. pertussis* for surveillance purposes, and to further explore the role of genetic adaptation. Developing new vaccine candidates, including allelic variants of ACV antigens present in the current circulating population, as well as exploring novel candidates, requires attention.

## Supplementary Information

Below is the link to the electronic supplementary material.

Supplementary file1 (DOCX 16 KB)

Supplementary file2 (DOCX 17 KB)

Supplementary file3 (PPTX 92 KB)

Supplementary file4 (PPTX 93 KB)

Supplementary file5 (XLSX 37 KB)

Supplementary file6 (XLSX 29 KB)

## Data Availability

The whole genome sequences generated and analyzed during the current study are available in European Nucleotide Archive (ENA) under the accession numbers ERS7736033 to ERS7736212.
