# Supplementary material for: Evolution of Bordetella pertussis in the acellular vaccine era in Norway, 1996 to 2019
Source: Eur J Clin Microbiol Infect Dis. 2022 May 11;41(6):913–24. doi: 10.1007/s10096-022-04453-0 (PMC9135841; doi:10.1007/s10096-022-04453-0)
Supplement: Supplementary file 1 — Supplementary file1 (DOCX 16 KB) [file 10096_2022_4453_MOESM1_ESM.docx]

**Supplementary**

**Evolution of *Bordetella pertussis* in the acellular vaccine era in Norway, 1996 to 2019**

Lin T. Brandal^1,2^, Didrik F. Vestrheim^1^, Torbjørn Bruvik^1^, Ragnhild B. Roness^1^, Martha L. Bjørnstad^1^, Margrethe Greve-Isdahl^1^, Anneke Steens^1^, Ola B. Brynildsrud^1,3^

^1^Norwegian Institute of Public Health, Oslo, Norway

^2^European Program for Public Health Microbiology Training (EUPHEM), European Centre for Disease Prevention and Control, (ECDC), Stockholm, Sweden

^3^Norwegian University of Life Science, Ås, Norway

Correspondence: Lin Thorstensen Brandal ([lin.thorstensen.brandal@fhi.no](mailto:lin.thorstensen.brandal@fhi.no))

**Figure S1**. Allelic profiles of acellular vaccine (ACV) antigens in Norwegian *B. pertussis* isolates, 1996-2019, and their association with alterations in the childhood immunisation programme in Norway. The whole cell vaccine (WCV) was used from 1952 until 1998. In 1998 ACV was introduced and in 2006/2007 a booster dose for 7 years old children was implemented. A second booster dose was introduced in the school year 2012/2013 for 15 years old adolescents. Seven different allelic profiles were identified among the Norwegian *B. pertussis* isolates. Allelic profile C (*ptxA1, ptxP1, prn2, fim2-1, fim3-1, fhaB1)* was common prior to implementation of ACV, whereas profile A2 (*ptxA1, ptxP3, prn2, fim2-1, fim3-2, fhaB7)* was frequently seen in the years between 1998-2003. From 2003 until 2007 allelic profile A1 (*ptxA1, ptxP3, prn2, fim2-1, fim3-2, fhaB1)* was most common, but after 2007 profile B (*ptxA1, ptxP3, prn2, fim2-1, fim3-1, fhaB1)* dominated among Norwegian *B. pertussis* isolates. In 2010 and 2011 no *B. pertussis* isolates or PCR positive samples were received at the National Reference Laboratory for pertussis at Norwegian Institute of Public Health.

**Figure S2**. Mutations within the *prn* gene in Norwegian *B. pertussis* isolates, 1996-2019, and their association with alterations in the childhood immunisation programme in Norway. In total, 17% (30/180) of the isolates showed mutations withinin the *prn* gene. These mutations were not present in isolates prior to introduction of the ACV, but were more common in the more recent years. In 2019 60% (6/10) of the isolates showed mutations within *prn*.

**Table S1**. Overview of Norwegian *B. pertussis* isolates (n=180) included in the study, 1996-2019.

**Table S2**. Overview of global *B. pertussis* sequences (n=371) included in the study, 1936-2016.
