# Supplementary material for: Evolution of Bordetella pertussis in the acellular vaccine era in Norway, 1996 to 2019
Source: Eur J Clin Microbiol Infect Dis. 2022 May 11;41(6):913–24. doi: 10.1007/s10096-022-04453-0 (PMC9135841; doi:10.1007/s10096-022-04453-0)
Supplement: Supplementary file 3 — Supplementary file3 (PPTX 92 KB) [file 10096_2022_4453_MOESM3_ESM.pptx]

## Slide 1
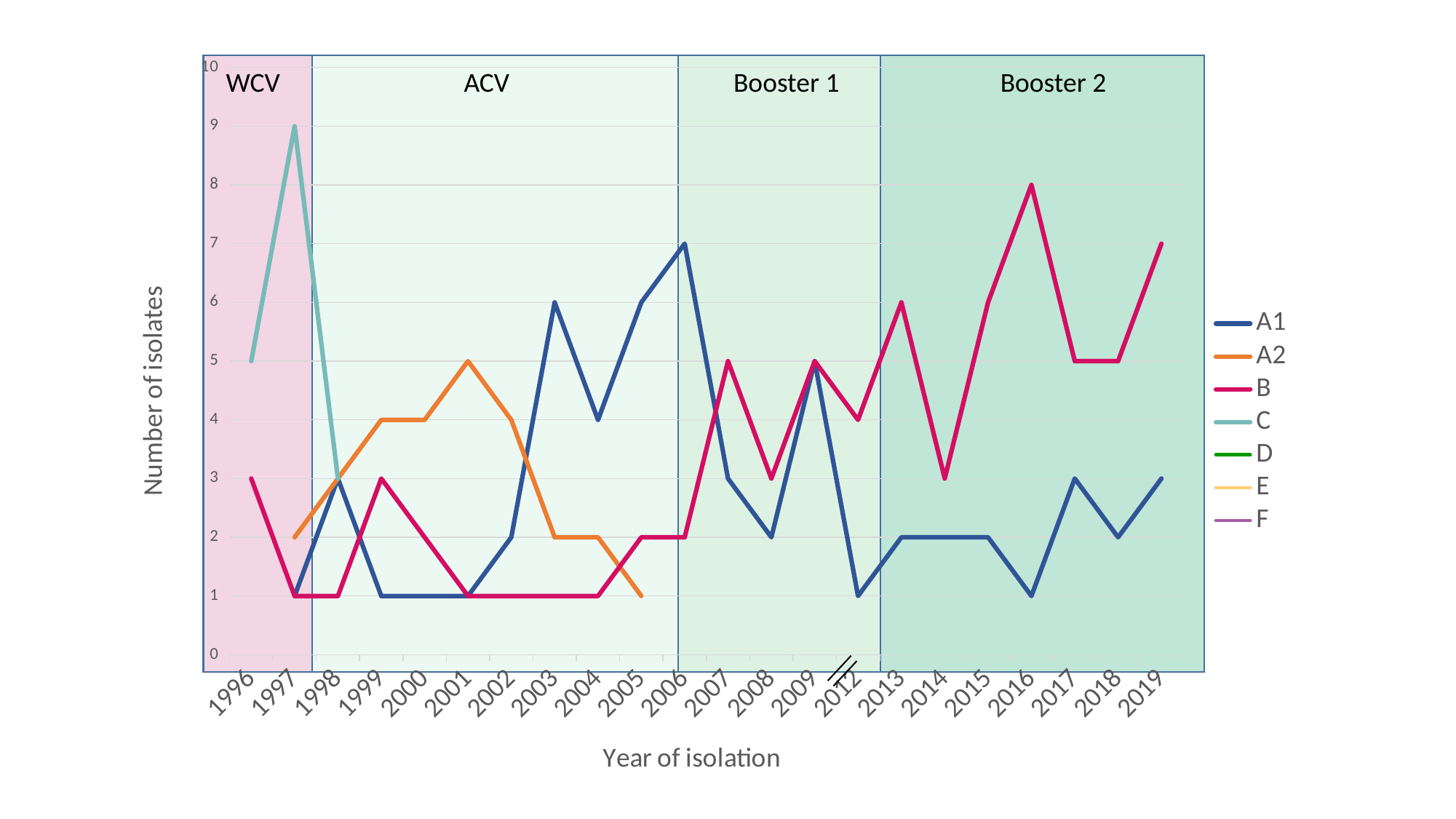

### Chart
| Category | A1 | A2 | B | C | D | E | F |
|---|---|---|---|---|---|---|---|
| 1996 | None | None | 3.0 | 5.0 | None | None | None |
| 1997 | 1.0 | 2.0 | 1.0 | 9.0 | None | 1.0 | None |
| 1998 | 3.0 | 3.0 | 1.0 | 3.0 | None | None | None |
| 1999 | 1.0 | 4.0 | 3.0 | None | None | None | None |
| 2000 | 1.0 | 4.0 | 2.0 | None | None | None | None |
| 2001 | 1.0 | 5.0 | 1.0 | None | None | None | None |
| 2002 | 2.0 | 4.0 | 1.0 | None | None | None | None |
| 2003 | 6.0 | 2.0 | 1.0 | None | None | None | None |
| 2004 | 4.0 | 2.0 | 1.0 | None | None | None | 1.0 |
| 2005 | 6.0 | 1.0 | 2.0 | None | 1.0 | None | None |
| 2006 | 7.0 | None | 2.0 | None | None | None | None |
| 2007 | 3.0 | None | 5.0 | None | None | None | None |
| 2008 | 2.0 | None | 3.0 | None | None | None | None |
| 2009 | 5.0 | None | 5.0 | None | None | None | None |
| 2012 | 1.0 | None | 4.0 | None | None | None | None |
| 2013 | 2.0 | None | 6.0 | None | None | None | None |
| 2014 | 2.0 | None | 3.0 | None | None | None | None |
| 2015 | 2.0 | None | 6.0 | None | None | None | None |
| 2016 | 1.0 | None | 8.0 | None | None | None | None |
| 2017 | 3.0 | None | 5.0 | None | None | None | None |
| 2018 | 2.0 | None | 5.0 | None | None | None | None |
| 2019 | 3.0 | None | 7.0 | None | None | None | None |
WCV
ACV
Booster 1
Booster 2
