# Supplementary material for: Evolution of Bordetella pertussis in the acellular vaccine era in Norway, 1996 to 2019
Source: Eur J Clin Microbiol Infect Dis. 2022 May 11;41(6):913–24. doi: 10.1007/s10096-022-04453-0 (PMC9135841; doi:10.1007/s10096-022-04453-0)
Supplement: Supplementary file 4 — Supplementary file4 (PPTX 93 KB) [file 10096_2022_4453_MOESM4_ESM.pptx]

## Slide 1
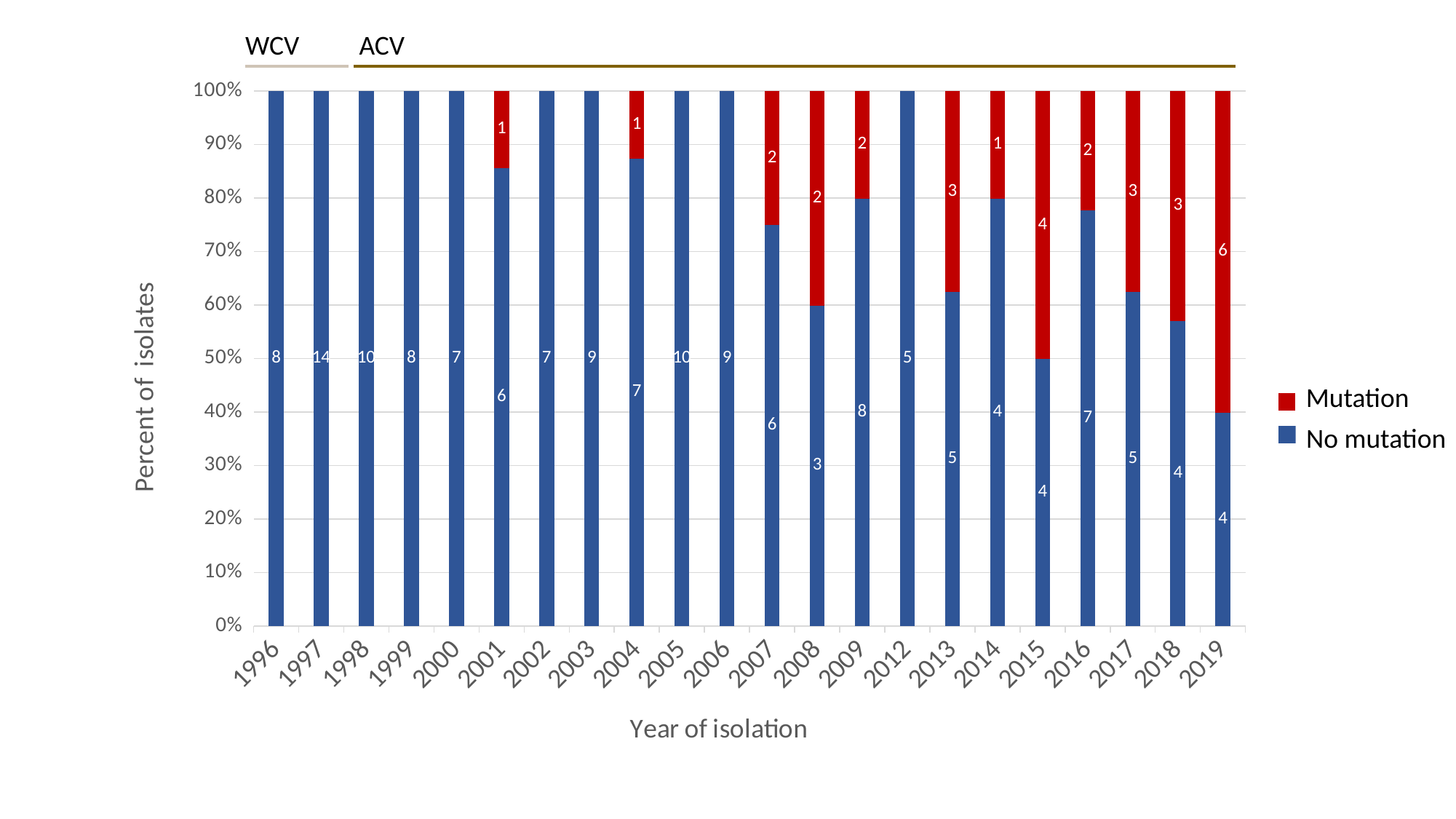

WCV
ACV
### Chart
| Category | 0 | 1 |
|---|---|---|
| 1996 | 8.0 | None |
| 1997 | 14.0 | None |
| 1998 | 10.0 | None |
| 1999 | 8.0 | None |
| 2000 | 7.0 | None |
| 2001 | 6.0 | 1.0 |
| 2002 | 7.0 | None |
| 2003 | 9.0 | None |
| 2004 | 7.0 | 1.0 |
| 2005 | 10.0 | None |
| 2006 | 9.0 | None |
| 2007 | 6.0 | 2.0 |
| 2008 | 3.0 | 2.0 |
| 2009 | 8.0 | 2.0 |
| 2012 | 5.0 | None |
| 2013 | 5.0 | 3.0 |
| 2014 | 4.0 | 1.0 |
| 2015 | 4.0 | 4.0 |
| 2016 | 7.0 | 2.0 |
| 2017 | 5.0 | 3.0 |
| 2018 | 4.0 | 3.0 |
| 2019 | 4.0 | 6.0 |Mutation
No mutation
